# Prostate Cancer Metastasis to the Pituitary Gland Manifesting as Corticosteroid Withdrawal, and the Impact of the Switch from Prednisone to Dexamethasone on Survival Time

**DOI:** 10.3390/curroncol28060365

**Published:** 2021-10-24

**Authors:** Okeroghene Ataikiru, Mahmoud Abdelsalam, Mrudula Avileli, Trina Hynes

**Affiliations:** 1 Oncology Clinical Trials, Horizon Health Network—The Moncton Hospital, Moncton, NB E1C 6Z8, Canada; Mrudula.Avileli@Horizonnb.ca (M.A.); Trina.Hynes@Horizonnb.ca (T.H.); 2 Oncology and Hematology Division, Horizon Health Network—The Moncton Hospital, Moncton, NB E1C 6Z8, Canada; Dr.Mahmoud.Abdelsalam@Horizonnb.ca

**Keywords:** prostate cancer, pituitary gland metastasis, corticosteroid, corticosteroid withdrawal, corticosteroid switch, survival time

## Abstract

Despite improvements in the diagnosis and treatment of cancers, the incidence of pituitary metastasis has increased. Prostate cancer metastasis to the pituitary, however, is rare, and these tumors usually grow rapidly. They are also more likely to be located in the posterior pituitary, and the presenting symptoms are often nonspecific, which makes early diagnosis challenging. The management of this condition is usually multidisciplinary, and requires careful assessment and decision making. We present a case of a patient who developed prostate cancer metastasis to the pituitary. In this report, we show that patients with prostate cancer on corticosteroid therapy who develop withdrawal symptoms or other endocrine symptoms should be assessed for pituitary and other brain metastasis. This case report also discusses the impact of switching from prednisone and abiraterone to dexamethasone and abiraterone. Our report shows that patients on abiraterone and prednisone whose PSA has increased, but who have no radiologic progression, may have their PSA controlled and thereby improved survival time when they are switched to abiraterone and dexamethasone.

## 1. Introduction

Though the diagnosis and treatment of cancers have improved, the incidence of pituitary metastasis has increased [1,2]. However, rarely do pituitary gland metastases originate from a prostate primary tumor; if they do, these metastases are more likely to be found in the posterior pituitary [1].

Additionally, switching patients with metastatic castrate-resistant prostate cancer who have biochemical progression with or without radiological progression from abiraterone acetate and prednisone to abiraterone acetate and dexamethasone at progression can improve their survival time [3]. Romero-Laorden et al. (2018) reported a median radiological progression-free survival of 11.8 months for patients who had been offered this switch. Although this switch is effective, patients should be monitored for adverse events such as hyperglycemia and muscle weakness [3].

We report a case of a patient with prostate cancer who underwent a switch from abiraterone acetate and prednisone to abiraterone acetate and dexamethasone when he progressed on the latter, and this improved his survival time. This patient also developed the symptoms of tumor metastasis to the pituitary gland that mimicked corticosteroid (dexamethasone) withdrawal.

## 2. Case Presentation

A 62-year-old male with a history of type II diabetes and obesity presented to the hospital because his prostate-specific antigen (PSA) level increased from 5.4 to 54.87 ng/mL. Prostate examination identified a nodule on the patient’s left side occupying the entire base of the midsection, and his prostate size was 35 g. A biopsy led to the diagnosis of infiltrating prostate adenocarcinoma with a Gleason score of 9 in May 2010. Computed tomographic scans of the abdomen and pelvis, and bone scans showed that there was metastasis to the bones in the lower spine, hip bone, and femur. The patient was put on a luteinizing hormone releasing hormone (LHRH) agonist (leuprolide (Eligard)) and an antiandrogen (bicalutamide (Casodex)). By January 2011, his PSA level was down to 0.06 ng/mL.

In June 2012, the patient presented to the emergency department with retention. Cystoscopy showed dynamic prostatic obstruction. The patient underwent transurethral resection of the prostate (TURP) to relieve this obstruction in July 2012. The patient also underwent a bilateral orchiectomy, failed another line of treatment, and then was started on abiraterone acetate (Zytiga) 1000 mg daily with prednisone 5 mg twice a day in June 2013 as first-line treatment for castrate-resistant metastatic prostate cancer. His PSA level improved to 0.66 ng/mL by September 2013.

The patient’s PSA level began to rise again and by April 2015, it was 12.54 ng/mL. He was switched from prednisone to dexamethasone 0.5 mg bid in May 2015. This brought his PSA level down to 0.45 ng/mL in September 2015. In October 2015, the patient began to experience side effects of the treatment, including uncontrolled diabetes, increased blood pressure, and trouble sleeping; these side effects were managed, and the patient continued his treatment. In December 2016, the patient began to develop a puffy moonlike face and cushingoid appearance. These symptoms were monitored, and in May–June 2017, the patient was taken off abiraterone acetate. Dexamethasone was also tapered from 0.5 mg bid to 0.5 mg once daily for 10 days. The patient developed withdrawal symptoms in July–August 2017 as he was being tapered off dexamethasone. The symptoms that he presented with included confusion, weight loss, headache, muscle and joint pain, weakness, nausea, fever, increased respiratory rate, and shortness of breath. The presentation of these withdrawal symptoms was surprising because the patient had been on a very low dose of dexamethasone. He was, therefore, put on 15 mg prednisone; the dose was then tapered down to 5 mg a day. When his condition had improved, he was switched to enzalutamide in September 2017, but he developed PSA and bone progression. Enzalutamide was stopped in December 2017, and the patient was started on docetaxel chemotherapy with denosumab and prednisone in January 2018.

**Figure 1:**
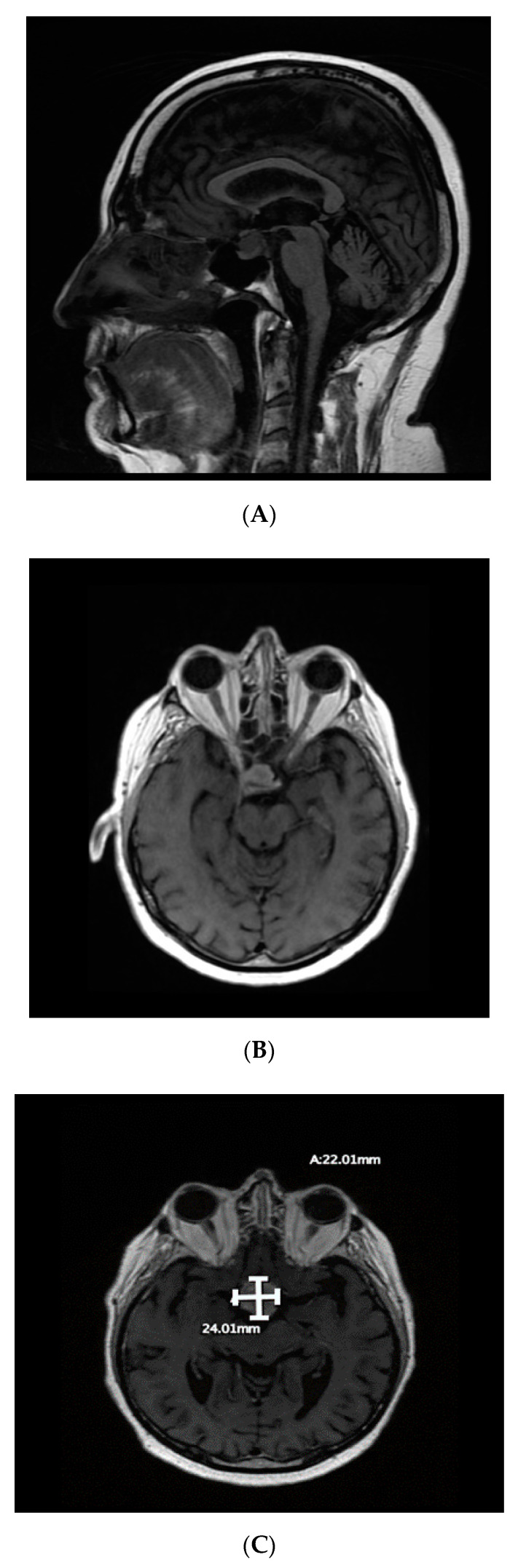
(**A**,**B**) Preoperative magnetic resonance imaging of the brain (June 2018) revealing a right sellar mass approximately 2.12 × 1.4 × 1.2 cm in size (A: sagittal view; B: axial view); (**C**) imaging during surgery in September 2018 showed that the mass had increased in size.

**Figure 2:**
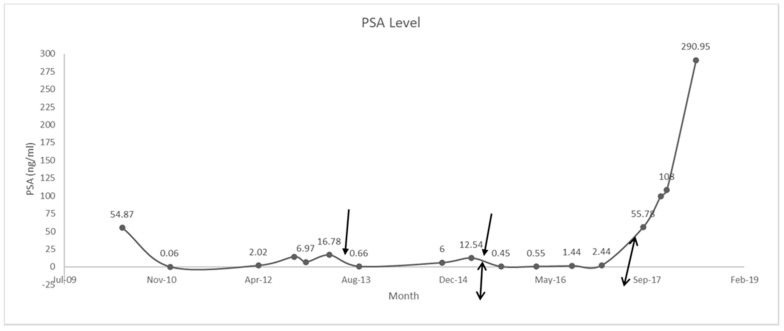
Dynamics of patient’s PSA levels. One-sided arrows, start and end of prednisone treatment (June 2013–May 2015). Double-sided arrows, start and end of dexamethasone treatment (May 2015–August 2017). Switch from prednisone to dexamethasone enabled the patient to achieve progression-free survival of an additional 28 months.

The patient developed a headache in May 2018. Brain magnetic resonance imaging (MRI) in June 2018 showed a new lesion in the right sellar region measuring 2.12 × 1.4 × 1.2 cm, extending to the suprasellar space and rising from the infundibulum and/or posterior pituitary gland (Figure 1). Two differential diagnoses were considered: prostate metastasis to the pituitary sella and incidental pituitary nonsecreting adenoma. The patient’s PSA level had then risen to 290 ng/mL (Figure 2), which suggested that the first option was the more likely diagnosis.

**Figure 3:**
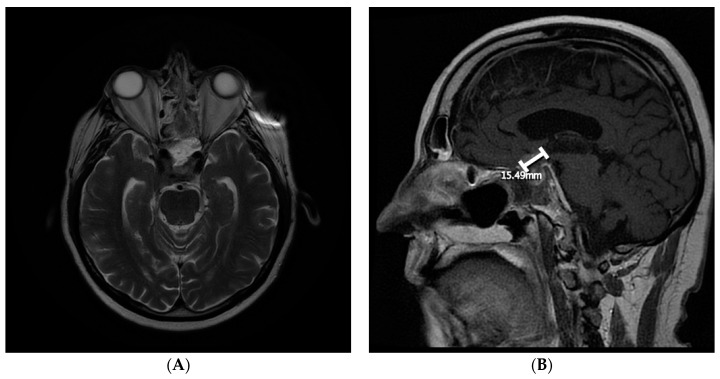
Follow-up postsurgical magnetic resonance imaging of the brain (September 2018) revealing subtotal surgical excision of the sellar metastatic lesion. (**A**) Axial view; (**B**) sagittal view.

By August 2018, the patient had developed bitemporal hemianopsia and decreased visual acuity, which progressed to complete visual loss. Transnasal transsphenoidal endoscopic resection of the mass was performed in September 2018, and navigation MRI performed during surgery revealed that the initial lesion had doubled in size, and that there was another right temporal lesion. Surgical excision was subtotal (Figure 3). Final pathology results indicated that the most likely diagnosis was metastatic adenocarcinoma, consistent with a prostatic primary tumor (sellar tumor). After surgery, more brain lesions were identified in the hypothalamus extending into the right basal ganglia and a dural base lesion.

The patient developed central diabetes insipidus during recovery from the transsphenoidal surgery. He was referred for stereotactic radiosurgery (SRS) to control his disease and symptoms. The patient could only receive part of the SRS treatment because of the proximity of the lesions to the optic nerve. His symptoms improved somewhat after the SRS, but his vision was not significantly restored. Systemic progression was also identified, but the patient declined any further treatment. The patient passed away in April 2019.

## 3. Discussion

Switching patients with metastatic castrate-resistant prostate cancer from abiraterone acetate and prednisone to abiraterone acetate and dexamethasone at progression can give patients more time [3]. This switch gave our patient an additional 28 months and it enabled chemotherapy to be delayed in this patient; he might have had even more time if he had not developed withdrawal symptoms. Romero-Laorden et al. (2018) reported a radiological progression-free survival of 11.8 months for patients who had been provided with this change in treatment. Our patient was progression-free for more than double this time before his withdrawal episode. Our patient also experienced a 96% reduction in his PSA level within 4 months of the switch from prednisone to dexamethasone. Although this treatment option is effective, patients should be monitored for adverse events related to the use of corticosteroids such as hyperglycemia, hypertension, and muscle weakness [3]. Our patient had his diabetes go out of control, he developed high blood pressure, and he developed a cushingoid facial appearance in response to the corticosteroid therapy.

Between 0.14% and 0.36% of intracranial metastases are in the pituitary [1,2]. Intracranial metastasis of prostate adenocarcinoma is uncommon, occurring in only 0.6–4.4% of cases [4]. The most common origins of pituitary metastases are breast cancer (9.3 times more than from other cancers) and lung cancer [1,5]. Pituitary metastasis usually occurs when patients are in their sixth decade of life, as was seen in our patient; it is more likely to be found in the posterior pituitary and often results in diabetes insipidus [1].

Our patient’s metastatic lesion was in the infundibulum and posterior pituitary, and he developed diabetes insipidus, which was controlled by desmopressin and fluid restriction. However, our patient’s diabetes insipidus could have been a postsurgical complication.

The posterior pituitary is in contact with the sellar bone, and any tumor that usually metastasizes to the bone, such as prostate cancer, can spread to this area of the pituitary gland [6]. Our patient had numerous bone metastases, including in the lower spine, left pelvic bone, and femur. He, therefore, was at risk for pituitary metastasis with sellar osseous involvement.

Metastatic tumors in the pituitary usually grow more rapidly than primary tumors do in this area [6,7]. For our patient, the pituitary metastatic tumor doubled in size within two months, consistent with this finding. The doubling in size of the tumor was also a confirmation of the diagnosis, as this increase in size is consistent with disease progression.

Most pituitary metastases are asymptomatic; only 7–10% are symptomatic, and these are usually at an advanced stage [1,4]. Other authors estimated the percentage of patients with pituitary metastasis who develop symptoms to be at 2.5–18.2% [1]. The most common clinical presentations of pituitary metastasis are panhypopituitarism, diabetes insipidus, visual symptoms, and headache; some patients do not have any pituitary-related symptoms [1,2]. Diabetes insipidus and visual symptoms are prominent symptoms that point to pituitary metastasis in patients with a known malignancy [8]. Many of these symptoms are due to the compression and/or invasion of surrounding structures such as cranial nerves [9]. Our patient developed a headache, which was his presenting symptom. He then developed hemianopsia due to optic-nerve compression, which progressed to complete blindness, and he also developed diabetes insipidus after surgery.

The symptoms of pituitary metastasis are often nonspecific, and this makes early diagnosis difficult [9]. Our patient had developed symptoms that were diagnosed as steroid withdrawal, one year before the diagnosis of pituitary metastasis. However, these symptoms may have been the early effects of the pituitary metastasis affecting the hypothalamic–pituitary–adrenal (HPA) axis and manifesting as steroid withdrawal.

The withdrawal of corticosteroids after long-term use temporarily reduces each component of the HPA axis’ response to endogenous and exogenous stimuli [10]. Our patient, after 4 years of treatment with prednisone and dexamethasone, was being tapered off the latter when he developed what seemed like withdrawal symptoms. However, these symptoms may have been the early effects of pituitary metastasis manifesting as steroidal withdrawal.

The management of pituitary metastasis may depend on the course of the primary tumor, the patient’s symptoms, and the extent of the disease [7]. In addition, decisions about how aggressive the treatment should be are dependent on the patient’s prognosis [8]. Some treatments for pituitary metastasis are trans-sphenoidal surgery, radiotherapy (ideally stereotactic), chemotherapy, targeted therapy, hormone therapy, and pituitary hormone substitution therapy [1,2]. Surgery may confirm the diagnosis and decrease symptoms. However, the total resection of the tumor is usually difficult because of the invasiveness of these tumors, and it may not improve survival [1,2]. Our patient had received trans-sphenoidal surgery, which resulted in subtotal excision and confirmed that the pituitary lesion was a metastasis of a prostatic primary. The patient also received SRS therapy and desmopressin hormonal therapy.

Median survival after the diagnosis of pituitary metastasis is 6–10 months. This survival time depends on the histology of the primary cancer and the used treatment modality [1,2]. Our patient lived for approximately 10 months after diagnosis of pituitary metastasis.

Our report shows that, at progression, patients with metastatic castrate-resistant prostate cancer could benefit from a switch from abiraterone acetate and prednisone to abiraterone acetate and dexamethasone. In addition, improved treatment modalities may increase the survival time of patients with malignant tumors, increasing the possibility that they may live long enough to develop rare metastases. Treating physicians should hence closely observe the symptoms of their patients with malignant tumors, so that such rare metastatic tumors can be caught early, before their severity increases. In light of this, patients with prostate cancer who are on corticosteroid therapy and who develop withdrawal symptoms or other endocrine symptoms should be assessed for pituitary and other brain metastases.

## Data Availability

Data sharing is not applicable to this article as no datasets were generated or analysed during the current study.
